# Comparison of Oncologic Outcomes and Treatment-Related Toxicity of Carbon Ion Radiotherapy and En Bloc Resection for Sacral Chordoma

**DOI:** 10.1001/jamanetworkopen.2021.41927

**Published:** 2022-01-07

**Authors:** Yagiz U. Yolcu, Jad Zreik, Waseem Wahood, Atiq ur Rehman Bhatti, Mohamad Bydon, Matthew T. Houdek, Peter S. Rose, Anita Mahajan, Ivy A. Petersen, Michael G. Haddock, Safia K. Ahmed, Nadia N. Laack, Krishan Jethwa, Elizabeth B. Jeans, Reiko Imai, Shigeru Yamada, Robert L. Foote

**Affiliations:** 1 Mayo Clinic Neuro-informatics Laboratory, Mayo Clinic, Rochester, Minnesota; 2 Department of Neurologic Surgery, Mayo Clinic, Rochester, Minnesota; 3 Central Michigan University College of Medicine, Mount Pleasant; 4 Dr Kiran C. Patel College of Allopathic Medicine, Nova Southeastern University, Davie, Florida; 5 Department of Orthopedic Surgery, Mayo Clinic, Rochester, Minnesota; 6 Department of Radiation Oncology, Mayo Clinic, Rochester, Minnesota; 7 QST Hospital, National Institutes for Quantum and Radiological Science and Technology, Inageku, Chiba, Japan

## Abstract

**Importance:**

Maximal resection is the preferred management for sacral chordomas but can be associated with unacceptable morbidity. Outcomes with radiotherapy are poor. Carbon ion radiotherapy (CIRT) is being explored as an alternative when surgery is not preferred.

**Objective:**

To compare oncologic outcomes and treatment-related toxicity of CIRT and en bloc resection for sacral chordoma.

**Design, Setting, and Participants:**

Univariable logistic regression was performed to evaluate the association between treatment type and oncologic and toxicity outcomes in this retrospective cohort study. Nearest-neighbor propensity score matching was used to match the CIRT cohort with the en bloc resection cohort and 10 National Cancer Database (NCDB) cohorts separately, with the objective of obtaining more homogeneous cohorts when comparing treatments. Patient- and tumor-related characteristics from 2 institutional cohorts were collected for patients diagnosed with sacral chordomas between April 1, 1994, and July 31, 2017. The NCDB was queried for data on patients with sacral chordoma from January 1, 2004, to December 31, 2016, as a comparator in overall survival (OS) analyses. Data analysis was conducted from February 24, 2020, to January 16, 2021.

**Exposures:**

En bloc resection, incomplete resection, photon radiotherapy, proton radiotherapy, and CIRT.

**Main Outcomes and Measures:**

Overall survival was estimated using the Kaplan-Meier method and compared using the Cox proportional hazards model. Peripheral motor nerve toxic effects were scored using Common Terminology Criteria for Adverse Events, version 4.03.

**Results:**

A total of 911 patients were included in the study (NCDB: n = 669; median age, 64 [IQR, 52-74] years; 410 [61.3%] men; CIRT: n = 188; median age, 66 [IQR, 58-71] years; 128 [68.1%] men; en bloc surgical resection: n = 54; median age, 53.5 [IQR 49-64] years, 36 [66.7%] men). Comparison of the propensity score–matched institutional en bloc resection and CIRT cohorts revealed no statistically significant difference in OS (CIRT: median OS, 68.1 [95% CI, 44.0-102.6] months; en bloc resection: median OS, 58.6 [95% CI, 25.6-123.5] months; *P* = .57; hazard ratio, 0.71 [95% CI, 0.25-2.06]; *P* = .53). The CIRT cohort experienced lower rates of peripheral motor neuropathy (odds ratio, 0.13 [95% CI, 0.04-0.40]; *P* < .001). On comparison of the propensity score–matched NCDB cohorts with the CIRT cohort, significantly higher OS was found for CIRT compared with margin-positive surgery without adjuvant radiotherapy (CIRT: median OS, 64.7 [95% CI, 57.8-69.7] months; margin-positive surgery without adjuvant radiotherapy: median OS, 60.6 [95% CI, 44.2-69.7] months, *P* = .03) and primary radiotherapy alone (CIRT: median OS, 64.9 [95% CI 57.0-70.5] months; primary radiotherapy alone: 31.8 [95% CI, 27.9-40.6] months; *P* < .001).

**Conclusions and Relevance:**

These findings suggest that CIRT can be used as treatment for older patients with high performance status and sacral chordoma in whom surgery is not preferred. CIRT might provide additional benefit for patients who undergo margin-positive resection or who are candidates for primary photon radiotherapy.

## Key Points

**Question:**

How do the outcomes of carbon ion radiotherapy (CIRT) compare with the outcomes obtained with en bloc surgical resection for sacral chordoma?

**Findings:**

In this cohort study including 911 patients with sacral chordomas, CIRT provided similar tumor control and survival outcomes compared with en bloc surgery but with a lower rate of peripheral motor neuropathy.

**Meaning:**

These findings suggest that CIRT is useful treatment for older patients with high performance status and sacral chordoma in whom surgery is not preferred.

## Teaser

This cohort study examines the use of carbon ion radiotherapy as treatment vs en bloc surgical resection in patients with sacral chordoma.

## Introduction

Chordomas are aggressive, slow-growing tumors that arise from remnants of embryonic notochord along the craniospinal axis.^1^ They manifest at an incidence of 8.4 per 10 million with approximately 30% occurring in the sacrum.^2^ En bloc surgical resection with negative margins is considered the preferred treatment for local tumor control and cure.^3^ Attaining negative margins is often difficult owing to large tumor size and invasion of adjacent structures, risk of postoperative morbidity, and anatomic complexity.^4,5,6^ Postoperative radiotherapy can be administered as a means to limit tumor progression and recurrence.^7^ For technically and medically inoperable tumors and patient preference, primary radiotherapy is the mainstay of treatment. Outcomes are poor with conventional radiotherapy alone.^8^

As an alternative, carbon ion radiotherapy (CIRT) has been studied as a treatment for sacral chordomas.^9,10,11,12^ CIRT deposits a relatively low dose of sparsely ionizing, low linear energy transfer radiation when entering the body and a relatively high dose of densely ionizing, high linear energy transfer radiation at the end of its range within the tumor target, eliminating the exit dose to healthy organs (Bragg peak effect). This physical effect allows a higher dose to be targeted to the tumor while reducing the dose and risk of radiation-associated toxic effects on surrounding organs.

In contrast, conventional photon radiotherapy deposits the maximal dose on entering the body, which decreases as it passes entirely through the body, limiting the delivery of the most effective dose and exposing healthy organs to the effects of ionizing radiation.^13^ In addition, carbon ions have distinct biological properties (high linear energy transfer, low oxygen enhancement ratio with high relative biological effectiveness) that theoretically could increase the success of treatment for traditionally radioresistant tumors, such as chordomas.^14^

Currently, CIRT is used to treat sacral chordomas at a small number of institutions globally and is not available in the US. As a result, there is a paucity of studies evaluating differences in oncologic and functional outcomes between CIRT and more readily accessible radiotherapy modalities. Therefore, we performed this retrospective cohort study using a propensity score–matching approach to control for differences in important confounders. We compared overall survival (OS), progression-free survival (PFS), local recurrence, distant metastasis, peripheral motor nerve toxic effects, urinary retention, need for colostomy, and change in Functional Mobility Scale levels for patients with sacral chordoma undergoing CIRT with patients undergoing en bloc resection, with or without adjuvant radiotherapy, or primary conventional radiotherapy from institutional cohorts and a national database.

## Methods

### En Bloc Resection Cohort

**Figure 1.**
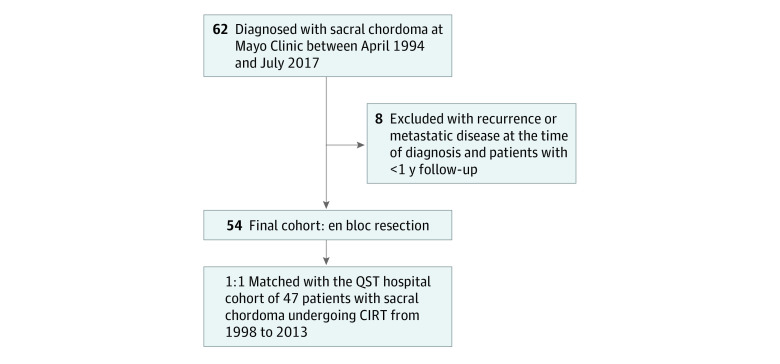
Flowchart for Selection of the Mayo Clinic En Bloc Resection Cohort Patients were identified using text search tools (Advanced Text Explorer and Mayo Data Explorer). Pathology laboratory reports were reviewed for confirmation of surgically resected or biopsied tumors. Imaging and clinical notes were used to identify additional chordoma diagnoses. Operative reports were reviewed to identify patients who underwent en bloc resection. Further medical records review was conducted for radiotherapy information. CIRT indicates carbon ion radiotherapy; QST, National Institutes for Quantum and Radiological Science and Technology, a national and international referral center for CIRT located in Chiba, Japan.

A retrospective review of medical records was conducted for patients diagnosed with chordoma of the sacrum between April 1, 1994, and July 31, 2017. Descriptions of eligibility criteria and a flowchart depicting the patient identification process are provided in Figure 1. Following eligibility screening, patients who underwent primary en bloc resection were included for analysis, hereafter referred to as the en bloc cohort. The institutional review board (IRB) of Mayo Clinic, Rochester, Minnesota, approved the study. Informed consent was waived because this retrospective review of medical records was considered minimal risk. The Strengthening the Reporting of Observational Studies in Epidemiology (STROBE) reporting guideline for observational studies was used in the revision of this article.^15^

### CIRT Cohort

**Figure 2.**
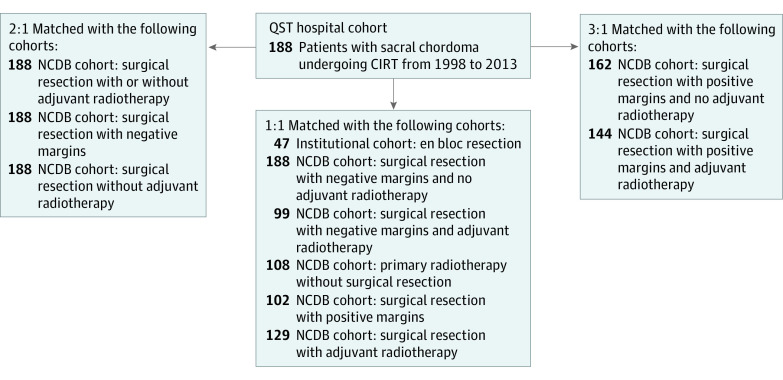
Flowchart for the Carbon Ion Radiotherapy (CIRT) Cohort NCDB indicates the National Cancer Database; QST, National Institutes for Quantum and Radiological Science and Technology, a national and international referral center for CIRT located in Chiba, Japan.

Following IRB approval at QST Hospital (National Institutes for Quantum and Radiological Science and Technology, a national and international referral center for CIRT located in Chiba, Japan), patients undergoing CIRT between January 1, 1998, and February 28, 2013, were screened for eligibility. Patients provided informed consent to participate in clinical trials and to use their records for research purposes. All patients had been enrolled on 1 of 2 treatment protocols for bone and soft-tissue sarcoma and were identified from a research database and were identified using *International Classification of Disease for Oncology, 2nd Edition*, and *International Classification of Disease for Oncology, 3rd Edition* (*ICD-O-3*) codes. The first protocol was from June 1, 1996, to December 31, 1999, and was performed as a phase 1/2 dose escalation clinical study. The second protocol began in 2000 as a phase 2 fixed-dose clinical study. The main eligibility criteria for the protocols were (1) the tumor was medically unresectable; (2) the tumor was histologically confirmed as sarcoma; (3) the tumor had not metastasized; (4) the tumor had not received prior radiotherapy, excluding radiation-associated sarcoma; and (5) the patient had no metal implantation to complicate treatment planning. Recurrence after resection and solitary metastasis were acceptable. A total of 637 patients were enrolled into these protocols during the study periods. Chordoma was confirmed with histologic testing in 223 patients and the sacrum was confirmed as the primary site in 210 patients. Twenty patients were found to have had recurrence after resection. Therefore, there were 190 patients with treatment-naive sacral chordoma; 2 were excluded. Patients with previously untreated chordoma confirmed by histopathologic testing who declined surgery or were diagnosed with unresectable disease were included in the present study. Patients were excluded if any of the following were present: (1) follow-up less than 1 year, (2) metastatic disease at the first visit, or (3) recurrent disease following the prior intervention for this diagnosis. A flowchart depicting the patient identification process is provided in Figure 2. Detailed information on eligibility and radiotherapy protocols has been published.^10^

### The National Cancer Database Cohorts

**Figure 3.**
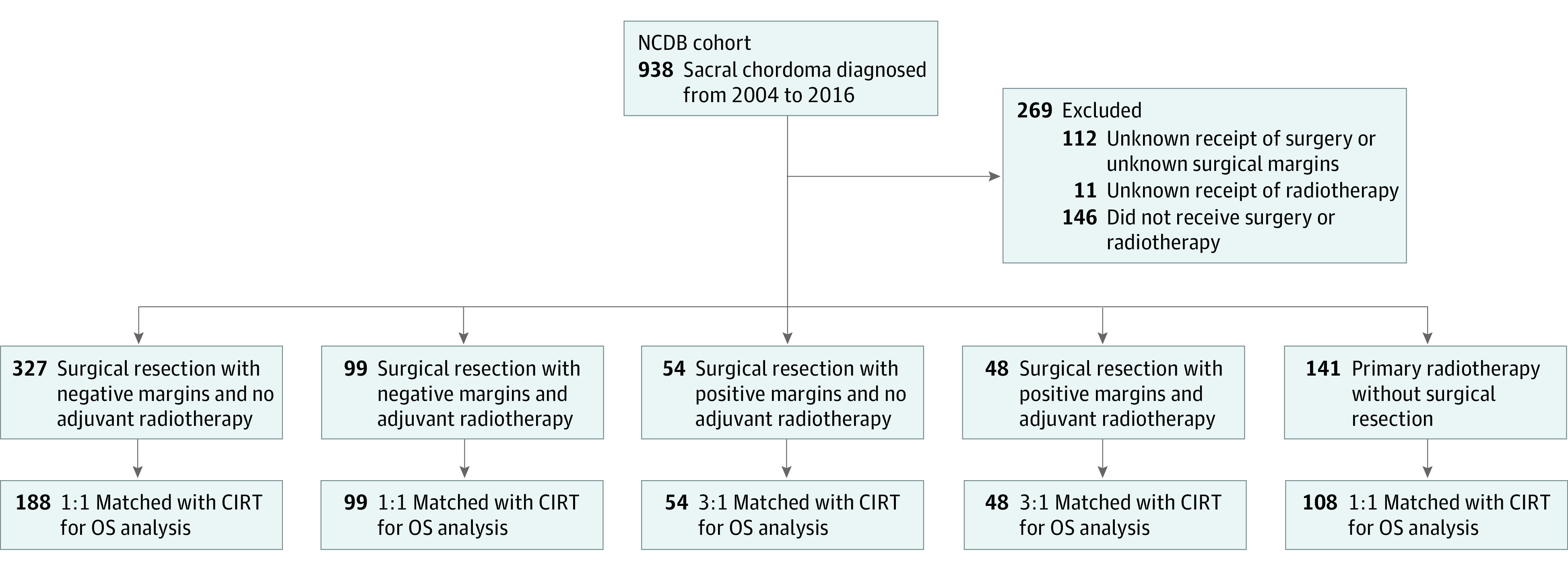
Flowchart for National Cancer Database (NCDB) Cohorts 1 to 5 CIRT indicates carbon ion radiotherapy; OS, overall survival.

**Figure 4.**
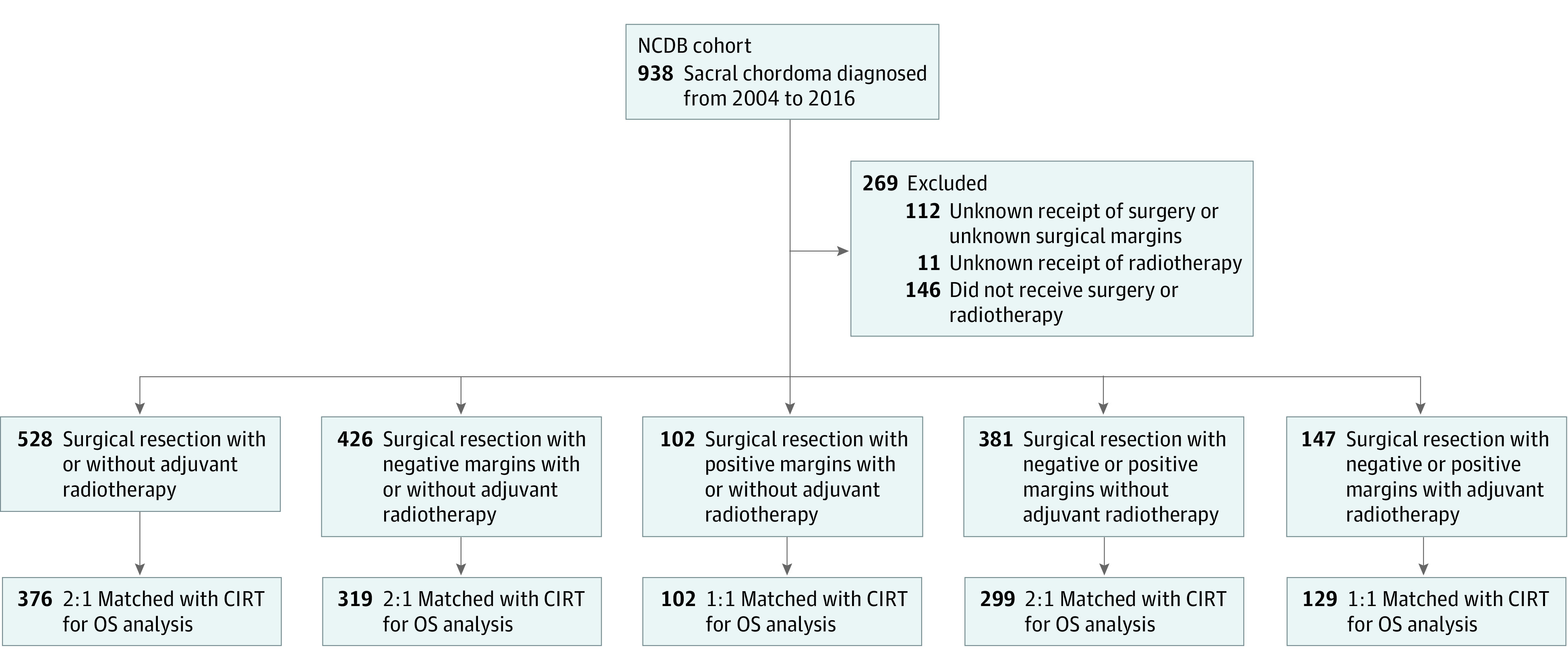
Flowchart for National Cancer Database (NCDB) Cohorts 6 to 10 CIRT indicates carbon ion radiotherapy; OS, overall survival.

The National Cancer Database (NCDB), a joint program between the Commission on Cancer and the American College of Surgeons, was developed as a means of quality improvement in oncology research and was queried for this study. Approximately 70% of all newly diagnosed cancer cases in the US are reported from over 1500 Commission on Cancer–accredited hospitals.^16^ All patient- and hospital-level data in the NCDB are deidentified; therefore, analysis of this data set was exempt from IRB approval as determined by the Mayo Clinic IRB. Sacral chordoma cases diagnosed from January 1, 2004, to December 31, 2016, were identified using *ICD-O-3* histologic codes designating chordoma (9370 and 9372). We further categorized tumors based on the *ICD-O-3* topographical codes designating pelvic bones, sacrum, coccyx, and associated joints (C41.4) or vertebral column (C41.2). Details on the type and extent of surgery and radiotherapy information are provided by institutions when data are submitted to the NCDB. Patients were excluded if there was no follow-up. Flowcharts depicting the patient identification process are provided in Figure 3 and Figure 4.

Patients in the NCDB were stratified by surgical margins (negative or positive) and treatment received (surgery, radiotherapy, or both) into the following 10 cohorts: (1) surgery, negative margins, no adjuvant radiotherapy; (2) surgery, negative margins, adjuvant radiotherapy; (3) surgery, positive margins, no adjuvant radiotherapy; (4) surgery, positive margins, adjuvant radiotherapy; (5) primary radiotherapy without surgery; (6) surgery, negative or positive margins, with or without adjuvant radiotherapy; (7) surgery, negative margins with or without adjuvant radiotherapy; (8) surgery, positive margins with or without adjuvant radiotherapy; (9) surgery, negative or positive margins, without adjuvant radiotherapy; and (10) surgery, negative or positive margins, with adjuvant radiotherapy (Figure 3 and Figure 4). Patients who received proton radiotherapy were included in our analysis. However, owing to the limited number of patients treated with protons, analysis and discussion of this treatment approach are provided in eAppendix 1 in the Supplement.

### Outcomes of Interest

The primary outcome of interest was OS for all cohorts. Secondary outcomes for the en bloc resection and CIRT cohorts included PFS, local recurrence, distant metastasis, peripheral motor nerve toxic effects (Common Terminology Criteria for Adverse Events version 4.03^17^) at the last follow-up or before recurrence, urinary retention at the last follow-up or before recurrence, colostomy required during or after treatment, and change in Functional Mobility Scale (FMS) scores^18^ from baseline to the last follow-up or before recurrence. Pretreatment Karnofsky Performance Scale^19^ and Eastern Cooperative Oncology Group Performance Status^20^ levels were recorded. For the NCDB cohorts, age, sex, radiotherapy modality, and OS were collected for analysis. Data on race were collected but not reported because all the patients from Japan were Japanese and race has not been reported to be associated with outcomes in patients with chordoma. Local recurrence, distant metastasis, toxic effects, and functional data were not available for the NCDB cohorts.

Standardized Medicare costs were obtained from the Mayo Clinic Cost Data Warehouse from 2007 to 2017 for the en bloc resection cohort and patients treated with primary proton radiotherapy. The details of this method have been published.^21^ In short, Medicare reimbursement was assigned to all professional services, the appropriate Medicare Cost Report cost-to-charge ratios were applied to charges for all hospital billed services, and all resulting costs were adjusted to 2017 US dollars with the gross domestic product implicit price deflator. For the CIRT cohort, Japanese episode reimbursement rates were obtained from the Japanese Ministry of Health for comparison.^22^ An exchange rate of 0.0096 US dollars per yen was used for conversion to US dollars.

### Statistical Analysis

Patients who received CIRT were propensity score matched to the en bloc resection cohort and each of the 10 NCDB cohorts to diminish potential biases from differences in patient baseline characteristics. Patient age, sex, baseline FMS score, highest level of tumor involvement, and tumor volume were used to match the CIRT cohort with the en bloc resection cohort (1:1 ratio). For the NCDB cohorts, only age and sex were used for matching as baseline functional status and specific tumor factors were not available. Depending on the number and characteristics of patients in each cohort, matching was performed in a 1:1, 2:1, or 3:1 ratio. We used nearest-neighbor propensity score matching. The standard mean difference was evaluated to ensure it approached 0 for each covariate for the matched data. Propensity scores were also checked for overlap, and they indicated a valid match for each comparison. The different matching ratios were chosen based on the number of patients in the 2 comparison groups and how similar patient characteristics were between groups to balance adequately controlling for confounders and maintaining the study sample size.

Patient characteristics and outcomes for each cohort are described using medians (IQRs) for continuous variables and frequencies with percentages for categorical variables. Descriptive statistics after matching for the comparison between the CIRT cohort, the en bloc resection cohort, and the 10 NCDB cohorts are presented. Baseline characteristics for the NCDB cohorts are also presented.

After matching, initial comparisons of values between CIRT and en bloc resection cohorts were made using the 2-sample *t* test for continuous variables and Pearson χ^2^ test for categorical variables. For these patients, univariate logistic regression was performed to evaluate the association between treatment modality (CIRT vs en bloc resection) and local recurrence, urinary retention, change in FMS, peripheral motor neuropathy, and colostomy. Similarly, the association between treatment modality (CIRT vs en bloc resection) and OS, PFS, and time to distant metastasis was evaluated using univariate Cox proportional hazards analyses. The proportional hazards assumption was verified to be met for each model via scaled Schoenfeld residuals. Patients with missing information were excluded from regression analyses. Kaplan-Meier analyses with log-rank tests were performed to compare OS, PFS, local recurrence, and distant metastasis between CIRT and en bloc resection cohorts.

The cumulative incidence for local recurrence and distant metastasis was calculated. In addition, Kaplan-Meier analyses with log-rank-tests were performed to compare OS between CIRT and each of the 10 NCDB cohorts. All analyses were performed using the R, version 3.6.1 statistical programming language (R Foundation for Statistical Computing). *P* values were 2-sided and a value <.05 was considered statistically significant. A multivariable analysis was not performed owing to the number of events, number of patients in each subgroup, and the lack of significant factors found on univariate analysis.

For the cost analysis, standardized Medicare charges were collected over a 2-year period and categorized into evaluation and management, imaging, radiotherapy, procedures, hospital services, and other costs for the en bloc resection cohort and a comparison group treated with primary proton radiotherapy at Mayo Clinic Rochester. Total mean 2-year costs and 2-year procedural charges, defined as the sum of procedure costs, radiotherapy costs, and hospital associated charges, were compared via the Mann-Whitney test. Data analysis was conducted from February 24, 2020, to January 16, 2021.

## Results

A total of 911 patients were included in the study (NCDB: n = 669; median age, 64 [IQR, 52-74] years; 410 [61.3%] men; 259 [38.7%] women; CIRT: n = 188; median age, 66 [IQR 58-71] years; 128 [68.1%] men; 60 [31.9%] women; en bloc surgical resection: n = 54; median age, 53.5 [IQR 49-64] years; 36 [66.7%] men; 18 [33.3%] women).

There was no statistically significant difference in OS, PFS, local recurrence, distant metastasis, urinary retention, or need for a colostomy between the en bloc resection cohort and the CIRT cohort in any of the analyses. There was a statistically significant higher rate and grade of peripheral motor neuropathy in the en bloc resection cohort. There was no statistically significant difference in OS between the NCDB surgery with negative margins cohort and the CIRT cohort.

### CIRT vs En Bloc Resection

eTable 1 in the Supplement contains the patient characteristics and outcomes for the unmatched CIRT and en bloc resection cohorts. After matching, a total of 94 patients were included in the analysis (47 patients in each cohort). The baseline characteristics for the 2 matched treatment cohorts are outlined in eTable 2 in the Supplement. There was no statistically significant difference in age, sex, tumor volume, or baseline FMS level. The difference in Eastern Cooperative Oncology Group performance status was statistically significant.

There was no significant difference in median OS (CIRT: 68.1 [95% CI, 44.0-102.6] months; en bloc resection: 58.6 [95% CI, 25.6-123.5] months; *P* = .57), median PFS (CIRT: 46.2 [95% CI, 33.3-75.7] months; en bloc resection: 40.7 [95% CI, 18.1-82.3] months; *P* = .55), local recurrence (CIRT: 9 of 47 [19.1%]; en bloc resection: 10 of 47 [21.3%]; *P* = .80), distant metastasis (CIRT: 14 of 47 [29.8%]; en bloc resection: 12 of 47 [25.5%]; *P* = .65), urinary retention (CIRT: no, 31 of 47; yes, 16 of 47; unknown, 0 of 47; en bloc resection: no, 20 of 47; yes, 16 of 47; unknown, 11 of 47; *P* = .34), or need for colostomy during or after treatment (CIRT: 9 of 47 [19.1%]; en bloc resection: 11 of 47 [23.4%]; *P* = .6) (eTable 2 in the Supplement). Most patients did not experience a change in their FMS scores from baseline to last follow-up or before recurrence; however, a higher percentage of patients had worse scores following treatment in the en bloc resection cohort, which was not statistically significant (34.0% vs 19.1%; *P* = .16). Patients who underwent en bloc resection had significantly higher rates of peripheral motor neuropathy and higher severity according to Common Terminology Criteria for Adverse Events, version 4.03 (CIRT: grade 0, 42 of 47; grade 1, 3 of 47; grade 2, 1 of 47; grade 3, 1 of 47; unknown, 0 of 47; en bloc resection: grade 0, 18 of 47, grade 1, 8 of 47; grade 2, 5 of 47; grade 3, 3 of 47; unknown, 13 of 47; *P* = .003). Additional outcomes are summarized in eTable 2 in the Supplement. The cumulative incidences of local recurrence and distant metastasis are shown in eFigure 1 in the Supplement.

Logistic regression and Cox proportional hazards analyses demonstrated similar results for the CIRT and en bloc resection propensity score–matched cohorts for the outcomes of interest (Table). The CIRT cohort had significantly lower odds of having peripheral motor neuropathy at the last follow-up (OR, 0.13; 95% CI, 0.04-0.40; *P* < .001). In addition, although statistical significance was not achieved, the CIRT cohort had lower odds of requiring a colostomy (OR, 0.78; 95% CI, 0.28-2.09; *P* = .62), developing local recurrence (OR, 0.88; 95% CI, 0.31-2.41; *P* = .80), and experiencing posttreatment urinary retention (OR, 0.65; 95% CI, 0.26-1.57; *P* = .34) compared with en bloc resection. On Cox proportional hazards analysis, there was no statistically significant difference in PFS (HR, 1.21; 95% CI, 0.61-2.42; *P* = .59), OS (HR, 0.71; 95% CI, 0.25-2.06; *P* = .53), or distant metastasis (HR, 1.44; 95% CI, 0.63-3.30; *P* = .39).

**Table.** CIRT vs En Bloc Resection Analysis

Outcome	HR or OR (95% CI)	*P* value
Cox proportional hazards		
Overall survival	0.71 (0.25-2.06)	.53
Progression-free survival	1.21 (0.61-2.42)	.59
Distant metastasis	1.44 (0.63-3.30)	.39
Univariate logistic regression		
Local recurrence	0.88 (0.31-2.41)	.80
Urinary retention	0.65 (0.26-1.57)	.34
Change in FMS score	2.41 (0.95-6.46)	.07
Peripheral motor neuropathy	0.13 (0.04-0.40)	<.001
Colostomy	0.78 (0.28-2.09)	.62

Abbreviations: CIRT, carbon ion radiotherapy; FMS, Functional Mobility Scale; HR, hazard ratio; OR, odds ratio.

### NCDB Unmatched Cohorts

eTable 3 in the Supplement contains the patient characteristics and outcomes for the unmatched NCDB cohorts. Median OS was highest for patients who underwent surgical resection with negative margins and received adjuvant radiotherapy (62.9 months); those who received radiotherapy alone had the lowest median OS (33.0 months).

### CIRT vs NCDB

After matching, a total of 669 patients were included in the analyses. The number of patients in each cohort and the baseline characteristics for the 11 matched treatment cohorts are outlined in eTable 4 in the Supplement. There was no statistically significant difference in age or sex.

Median OS was significantly greater in the CIRT cohort compared with the NCDB cohorts receiving primary radiotherapy (CIRT: 64.9 [95% CI, 57.0-70.5] months; primary radiotherapy: 31.8 [95% CI, 27.9-40.6] months; *P* < .001) or surgery with positive margins but no adjuvant radiotherapy (CIRT: 64.7 [95% CI, 57.8-69.7] months; surgery with positive margins but no adjuvant radiotherapy: 60.6 [95% CI, 44.2-69.7] months; *P* = .03) (eFigure 3A and 3B in the Supplement). The cohorts who underwent surgery with negative margins with adjuvant therapy (CIRT: 67.7 [95% CI, 57.3-76.5] months; surgery with negative margins with adjuvant radiotherapy: 62.9 [95% CI, 58.0-68.9] months; *P* = .83) (eFigure 3C in the Supplement) and without adjuvant therapy (CIRT: 62.2 [95% CI, 56.7-68.5] months; surgery with negative margins without adjuvant radiotherapy: 59.0 [95% CI, 49.7-72.2] months; *P* = .96) (eFigure 3D in the Supplement) adjuvant radiotherapy as well as surgery with positive margins and adjuvant radiotherapy (CIRT: 64.7 [95% CI, 40.8-89.0] months; surgery with positive margins and adjuvant radiotherapy: 51.6 [95% CI, 38.7-70.7] months; *P* = .19) (eFigure 3E in the Supplement) had similar median OS compared with the CIRT cohort. The cohort undergoing surgery with positive margins with or without adjuvant radiotherapy had lower median OS compared with the CIRT cohort (CIRT: 71.8 [95% CI, 64.3-82.0] months; surgery with positive margins with or without adjuvant radiotherapy: 55.0 [95% CI, 48.3-64.9] months; *P* = .02) (eFigure 4C in the Supplement). eTable 5 in the Supplement reports the fractions, dose, and biologically effective dose details for nonsurgical patients who were treated with primary photon radiotherapy, proton radiotherapy, or CIRT. eFigure 5 in the Supplement shows significantly higher OS rates for the matched cohort who received primary CIRT vs photon radiotherapy (CIRT: median OS, 46.0 [95% CI, 41.0-57.8] months; photon radiotherapy: median OS, 31.2 [95% CI, 26.7-40.9] months; *P* < .001) compared with the OS rates of the primary CIRT vs proton radiotherapy cohorts (CIRT: median OS, 65.5 [95% CI, 57.0-82.0] months; proton radiotherapy: median OS, 43,2 [95% CI, 30.9-50.6] months; *P* = .95).

Two-year standardized Medicare costs were available for 28 patients treated with en bloc resection and 15 patients treated with proton radiotherapy at Mayo Clinic (eTable 6 in the Supplement). Both mean (SD) total costs and total procedural costs were significantly higher for the en bloc resection cohort at $137 182 ($117 894) and $110 375 ($94 169) (*P* = .002) vs $68 066 ($60 567) and $53 699 ($43 052) (*P* < .01) for the proton group.

## Discussion

In this retrospective cohort study, a comparison of en bloc resection and CIRT for treatment of sacral chordomas did not reveal any statistically significant differences in OS, PFS, local tumor control, or distant metastases. However, the rate and grade of peripheral motor neuropathy at the last follow-up was significantly higher for en bloc resection. Compared with a national cohort of patients with sacral chordoma, CIRT was associated with a higher OS rate than primary radiotherapy and primary surgery with positive margins. There was no significant difference in OS between CIRT and surgery with negative margins. An exploratory cost analysis suggested that definitive proton radiotherapy or CIRT may be associated with significantly reduced global and procedural costs compared with en bloc resection, despite the cost of advanced technology (eAppendix 2 and eTable 6 in the Supplement).

Achieving a safe en bloc resection with negative margins remains the preferred treatment for sacrococcygeal chordomas.^23,24^ However, a number of circumstances may make achieving gross total resection infeasible.^5,25,26^ The earliest phase 1/2 trial reports on the safety and efficacy of CIRT showed 5-year survival and local control rates approaching 90% with favorable ambulatory function.^11,12^ Moreover, Wu et al^27^ evaluated the outcomes of CIRT for patients who did not undergo surgical resection and reported 2-year progression-free survival (80.4%) and local control (85.2%) rates.

The propensity score–matching comparison between the en bloc resection and CIRT cohorts in our study attempted to give a preliminary answer to this question using the existing data. Analysis of matched cohorts showed similar oncologic and toxic effects outcomes in general with lower peripheral motor neuropathy and cost in the CIRT group, thus further supporting CIRT as a potential alternative to surgery in certain populations. Patients in the CIRT cohort had larger tumors that were more invasive into adjacent pelvic organs and were considered unresectable owing to age, comorbidity, and quality-of-life and functional concerns of the surgeon and patient.

Another area of interest for the use of CIRT is as adjuvant radiotherapy following surgical resection. Although this question has not been answered, Uhl et al^28^ reported an OS rate of 100% and local control rate of 85% (median follow-up, 25 months) for 56 patients undergoing postoperative CIRT at a single institution. In our analyses, we found significantly lower OS for patients undergoing adjuvant radiotherapy following a margin-positive resection compared with primary CIRT, outlining a need for studies evaluating the use of CIRT as adjuvant radiotherapy compared with conventional radiotherapy with photons or protons.^29,30^

### Limitations

This study has limitations. One limitation is that uncontrolled confounding variables may have been present owing to the study’s retrospective design. Although we controlled for multiple factors in an attempt to ensure the cohorts were similar, there likely was residual confounding owing to unknown prognostic factors in this rare disease and the inclusion and exclusion criteria used. The en bloc resection sample was also relatively small compared with the CIRT cohort. Because the operations were performed between 1994 and 2017, there may have been substantial variability in procedural advances over time. Although the NCDB cohorts were larger, information on performance status, tumor volume, highest tumor level, recurrence, toxic effects, and distant metastasis at the time of diagnosis was not available. The data for the CIRT cohort were only available for patients who did not undergo surgery, preventing an assessment for the potential value of CIRT as an adjuvant therapy. An adjusted analysis based on clinic, hospital, or geographic region was not possible owing to CIRT and en bloc resection cohorts containing single institutional data. Cost analysis data were limited owing to a small number of patients and because follow-up costs for more than 2 years were not analyzed. However, we anticipate that the significant associations found in this multi-institutional analysis will guide future prospective comparisons of these important treatment modalities.

## Conclusions

These findings suggest that CIRT is a satisfactory treatment for cases in which resection is technically or medically inadvisable or patients prefer not to undergo the procedure. Moreover, the use of CIRT as adjuvant radiotherapy might provide additional benefit for patients who undergo subtotal resection.

## Notes

Supplement. **eFigure 1.** Cumulative Incidence for Local Recurrence and Distant Metastasis CIRT Compared to En Bloc Resection **eFigure 2.** Kaplan-Meier Plots Comparing CIRT and En Bloc Resection Cohorts After Propensity Score Matching **eFigure 3.** Kaplan-Meier Plots Comparing OS of CIRT to NCDB Cohorts After Matching for Age and Sex **eFigure 4.** Kaplan-Meier Plots Comparing OS of CIRT to NCDB Cohorts After Matching for Age and Sex **eTable 1.** Characteristics of CIRT vs En Bloc Resection Cohorts Prior to Matching **eTable 2.** Characteristics of Matched CIRT vs En Bloc Resection Cohorts **eTable 3.** Baseline Characteristics of NCDB Cohorts **eTable 4.** Characteristics and Overall Survival of Matched CIRT Cohort vs Each NCDB Cohort **eAppendix 1.** NCDB Proton Cohort Analysis **eFigure 5.** Kaplan-Meier Plots Comparing OS Between Matched CIRT and Primary Radiotherapy NCDB Cohorts **eTable 5.** Radiotherapy Treatment Factors by Modality for Unmatched NCDB Patients Who Underwent Primary RT and for Primary CIRT Patients **eAppendix 2.** Cost Analysis **eTable 6.** Two-Year Standardized Medicare Costs for En Bloc Resection and Proton Radiotherapy at Mayo Clinic From 2007 to 2016
eReferences
