# Supplementary material for: Comparison of Oncologic Outcomes and Treatment-Related Toxicity of Carbon Ion Radiotherapy and En Bloc Resection for Sacral Chordoma
Source: JAMA Netw Open. 2022 Jan 7;5(1):e2141927. doi: 10.1001/jamanetworkopen.2021.41927 (PMC8742192; doi:10.1001/jamanetworkopen.2021.41927)
Supplement: Supplement. — eFigure 1. Cumulative Incidence for Local Recurrence and Distant Metastasis CIRT Compared to En Bloc Resection eFigure 2. Kaplan-Meier Plots Comparing CIRT and En Bloc Resection Cohorts After Propensity Score Matching eFigure 3. Kaplan-Meier Plots Comparing OS of CIRT to NCDB Cohorts After Matching for Age and Sex eFigure 4. Kaplan-Meier Plots Comparing OS of CIRT to NCDB Cohorts After Matching for Age and Sex eTable 1. Characteristics of CIRT vs En Bloc Resection Cohorts Prior to Matching eTable 2. Characteristics of Matched CIRT vs En Bloc Resection Cohorts eTable 3. Baseline Characteristics of NCDB Cohorts eTable 4. Characteristics and Overall Survival of Matched CIRT Cohort vs Each NCDB Cohort eAppendix 1. NCDB Proton Cohort Analysis eFigure 5. Kaplan-Meier Plots Comparing OS Between Matched CIRT and Primary Radiotherapy NCDB Cohorts eTable 5. Radiotherapy Treatment Factors by Modality for Unmatched NCDB Patients Who Underwent Primary RT and for Primary CIRT Patients eAppendix 2. Cost Analysis eTable 6. Two-Year Standardized Medicare Costs for En Bloc Resection and Proton Radiotherapy at Mayo Clinic From 2007 to 2016 eReferences [file jamanetwopen-e2141927-s001.pdf]

Supplementary Online Content

Yolcu YU, Zreik J, Wahood W, et al. Comparison of oncologic outcomes and treatment-related toxicity of carbon ion radiotherapy and en bloc resection for sacral chordoma. *JAMA Netw Open*. 2022;5(1): e2141927. doi:10.1001/jamanetworkopen.2021.41927

eFigure 1. Cumulative Incidence for Local Recurrence and Distant Metastasis. CIRT Compared to En Bloc Resection

eFigure 2. Kaplan-Meier Plots Comparing CIRT and En Bloc Resection Cohorts After Propensity Score Matching

eFigure 3. Kaplan-Meier Plots Comparing OS of CIRT to NCDB Cohorts After Matching for Age and Sex

eFigure 4. Kaplan-Meier Plots Comparing OS of CIRT to NCDB Cohorts After Matching for Age and Sex

eTable 1. Characteristics of CIRT vs En Bloc Resection Cohorts Prior to Matching

eTable 2. Characteristics of Matched CIRT vs En Bloc Resection Cohorts

eTable 3. Baseline Characteristics of NCDB Cohorts

eTable 4. Characteristics and Overall Survival of Matched CIRT Cohort vs Each NCDB Cohort

eAppendix 1. NCDB Proton Cohort Analysis

eFigure 5. Kaplan-Meier Plots Comparing OS Between Matched CIRT and Primary Radiotherapy NCDB Cohorts

eTable 5. Radiotherapy Treatment Factors by Modality for Unmatched NCDB Patients Who Underwent Primary RT and for Primary CIRT Patients

eAppendix 2. Cost Analysis

eTable 6. Two-Year Standardized Medicare Costs for En Bloc Resection and Proton Radiotherapy at Mayo Clinic From 2007 to 2016

eReferences

This supplementary material has been provided by the authors to give readers additional information about their work.

© 2022 Yolcu YU et al. *JAMA Network Open.*

eFigure 1. Cumulative Incidence for (A) local recurrence and (B) distant metastasis. CIRT Compared to En Bloc Resection

.

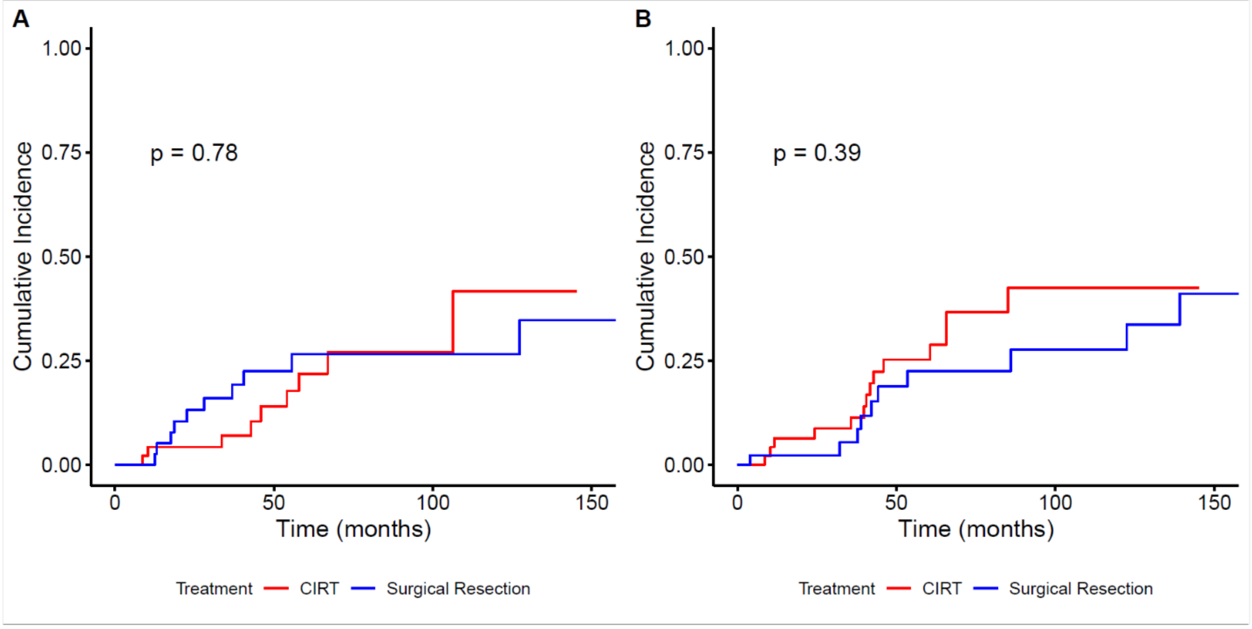

© 2022 Yolcu YU et al. *JAMA Network Open.*

eFigure 2. Kaplan-Meier plots comparing CIRT and en bloc resection cohorts after propensity score matching.

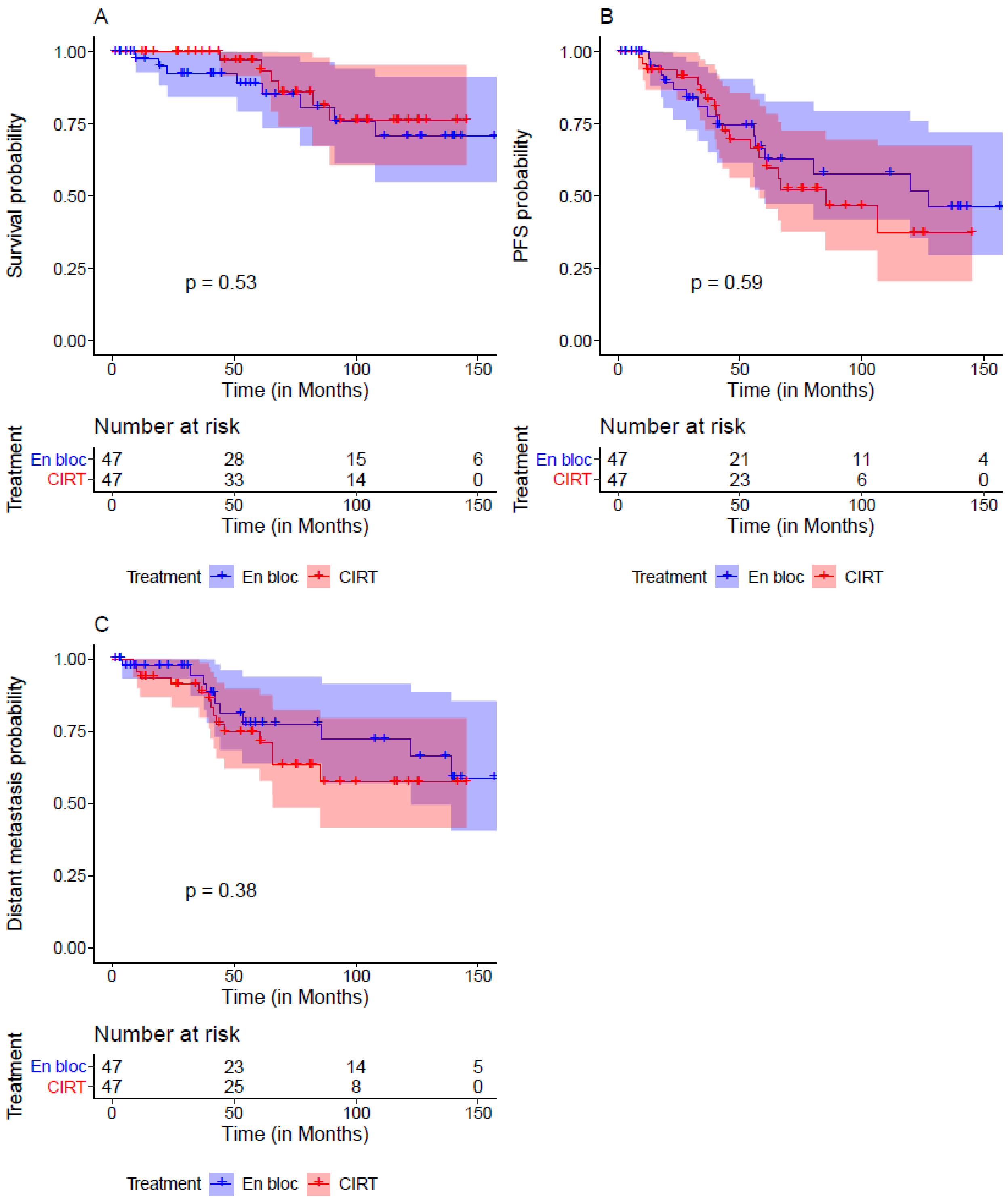

(A) OS, (B), PFS, and (C) distant metastasis.

© 2022 Yolcu YU et al. *JAMA Network Open.*

eFigure 3. Kaplan-Meier plots comparing OS of CIRT to NCDB cohorts after matching for age and sex.

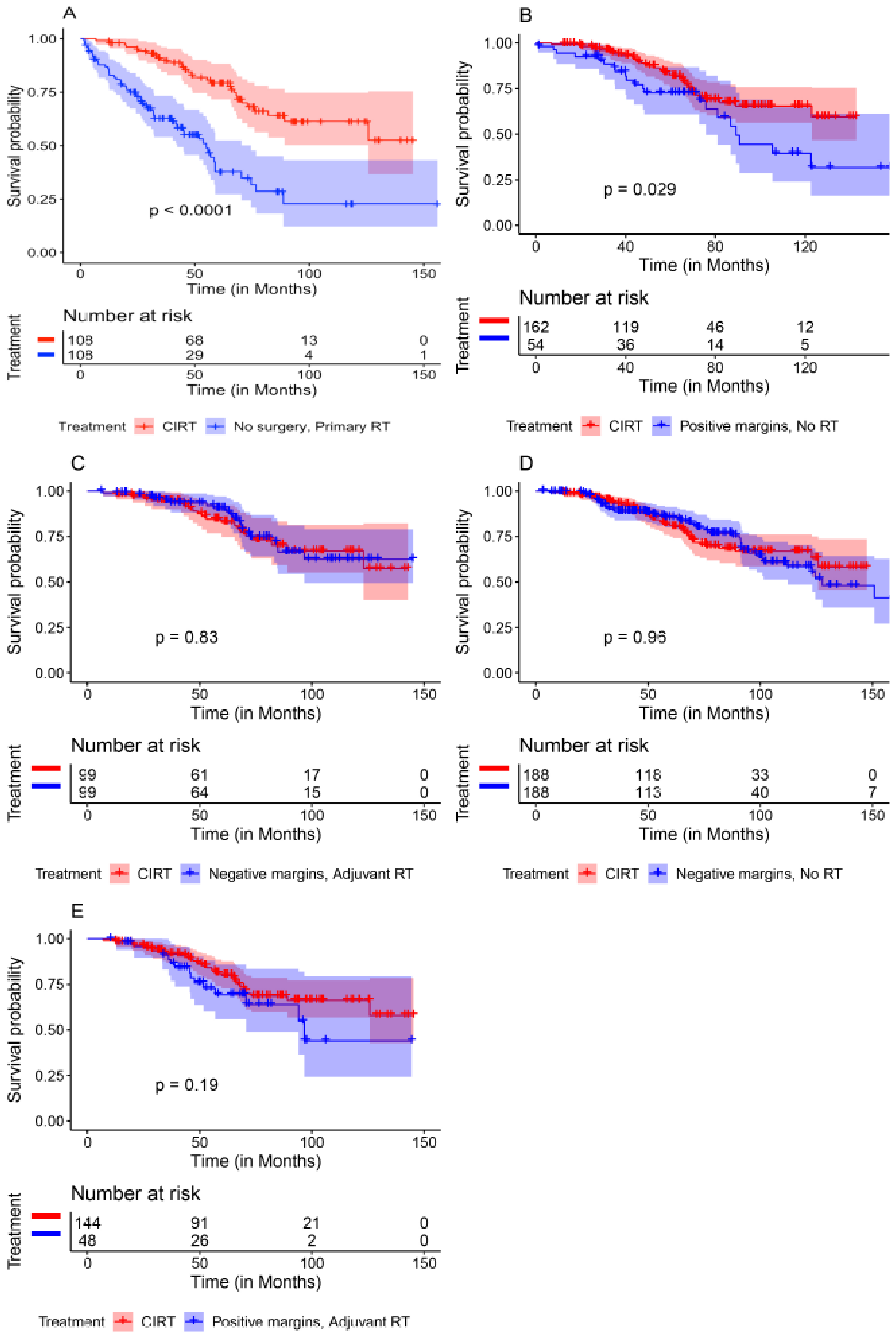

(A) no surgery, primary radiotherapy (RT) only, (B) positive margins, no adjuvant radiotherapy (RT), (C) negative margins, adjuvant radiotherapy (RT), (D) negative margins, no adjuvant radiotherapy (RT), (E) positive margins, adjuvant radiotherapy (RT).

© 2022 Yolcu YU et al. *JAMA Network Open.*

eFigure 4. Kaplan-Meier plots comparing OS of CIRT to NCDB cohorts after matching for age and sex.

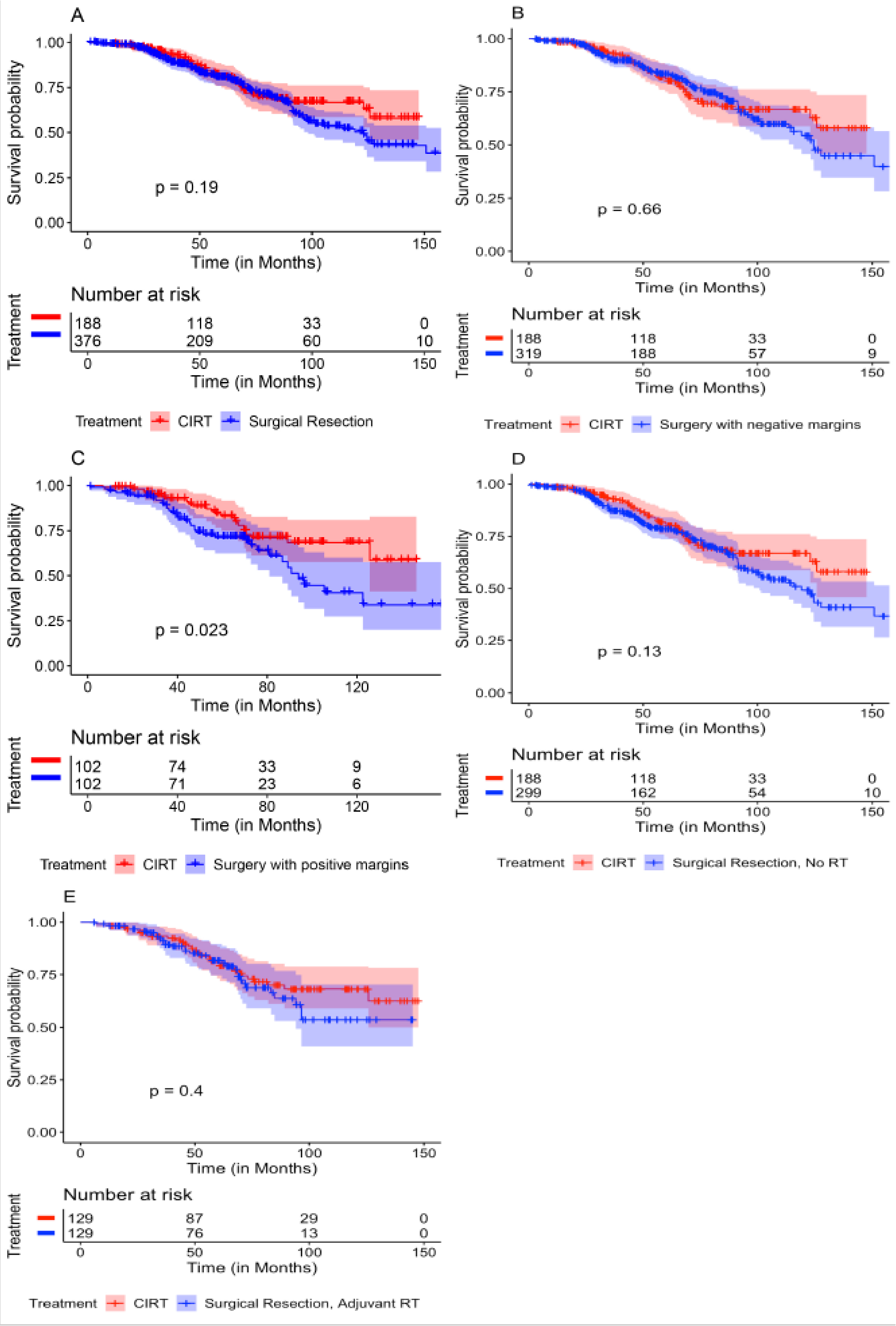

(A) surgical resection, positive or negative margins, with or without radiotherapy, (B) surgery with negative margins, with or without radiotherapy (C) surgery with positive margins, with or without radiotherapy (D) surgical resection, no radiotherapy (RT), (E) surgical resection, with postoperative radiotherapy (RT).

eTable 1. Characteristics of CIRT vs en bloc resection cohorts prior to matching

	CIRT (N=188)	En bloc Resection (N=54)	P-value
Age, median (IQR)	66 (58-71)	53.5 (49-64)	**<0.001**
Sex (male), n (%)	128 (68.1%)	36 (66.7%)	0.844
Baseline FMS, n (%)			**<0.001**
1	1 (0.5%)	0 (0.0%)	
2	5 (2.7%)	0 (0.0%)	
4	17 (9.2%)	1 (2.0%)	
5	53 (28.6%)	2 (4.1%)	
6	109 (58.9%)	46 (93.9%)	
Unknown	4	5	
Tumor size (cm^3), median (IQR)	344.6 (195.8-546.8)	181.1 (60.6-372.8)	0.838
Highest Level of Involvement, n (%)			**<0.001**
L5	11 (5.9%)	0 (0.0%)	
S1	49 (26.1%)	7 (13.0%)	
S2	77 (41.0%)	15 (27.8%)	
S3	38 (20.2%)	15 (27.8%)	
S4	13 (6.9%)	9 (16.7%)	
S5	0 (0.0%)	6 (11.1%)	
Coccyx	0 (0.0%)	2 (3.7%)	
Unknown	1	0	
Karnofsky Performance Scale, n (%)			0.100
100	3 (1.6%)	3 (6.4%)	
90	102 (54.3%)	27 (57.4%)	
80	52 (27.7%)	15 (31.9%)	
70	28 (14.9%)	2 (4.3%)	
60	3 (1.6%)	0 (0.0%)	
Unknown	1	7	
ECOG Performance Status, n (%)			**<0.001**
0	3 (1.6%)	33 (68.8%)	
1	158 (84.0%)	14 (29.2%)	
2	27 (14.4%)	1 (2.1%)	
Unknown	1	6	
Overall survival (months), median (95% CI)	62.2 (36.5-88.4)	55.7 (26.0-118.5)	0.337
Progression free survival (months), median (IQR)	41.2 (36.5-88.4)	55.7 (26.0-118.5)	0.651
Local recurrence, n (%)	41 (21.8%)	14 (25.9%)	0.525
Distant metastasis, n (%)	54 (28.7%)	16 (29.6%)	0.897
Site of distant metastasis, n (% of metastasized tumors)			0.820
Spinal/pelvis bone	18 (33.3%)	4 (25.0%)	
Lung	15 (27.8%)	5 (31.2%)	
Other	21 (38.9%)	7 (43.8%)	

© 2022 Yolcu YU et al. *JAMA Network Open.*

eTable 1. Characteristics of CIRT vs en bloc resection cohorts prior to matching (cont.)

	CIRT (N=188)	En bloc Resection (N=54)	P-value
**Peripheral motor nerve toxicity grade at last follow up prior to recurrence, n (%)***			**0.016**
0	147 (78.6%)	21 (55.3%)	
1	21 (11.2%)	9 (23.7%)	
2	15 (8.0%)	5 (13.2%)	
3	4 (2.1%)	3 (7.9%)	
Unknown	2	16	
**Urinary retention, n (%)**			0.907
No	105 (56.1%)	24 (57.1%)	
Yes	82 (43.9%)	18 (42.9%)	
Unknown	2	12	
**Colostomy, n (%)**	31 (16.5%)	14 (25.9%)	0.116
**FMS at last follow-up before recurrence, n (%)**			0.048
1	5 (2.7%)	3 (6.0%)	
2	6 (3.2%)	4 (8.0%)	
3	3 (1.6%)	1 (2.0%)	
4	32 (17.1%)	3 (6.0%)	
5	54 (28.9%)	8 (16.0%)	
6	87 (46.5%)	31 (62.0%)	
Unknown	2	4	
**Change in FMS, n (%)**			**0.037**
Improved	3 (1.6%)	1 (1.9%)	
Same	143 (77.7%)	32 (60.4%)	
Worse	38 (20.7%)	20 (37.7%)	
Unknown	4	1	
**Cause of death, n (%)**			NA
**Old age**	24 (12.7%)	NA	
**Infectious disease**	1 (0.5%)	NA	
**Cardiovascular disease**	3 (1.6%)	NA	
**Pneumonitis**	3 (1.6%)	NA	
**Other**	14 (7.4%)	NA	

CIRT: Carbon ion radiotherapy, FMS: Functional mobility scale
*CTCAE version 4.03, NA: Not available

© 2022 Yolcu YU et al. *JAMA Network Open.*

eTable 2. Characteristics of matched¥ CIRT vs en bloc resection cohorts

	CIRT (N=47)	En bloc Resection (N=47)	P-value	SMD
**Age, median (IQR)**	58 (46-66)	54 (49-64)	0.734	0.072
**Sex (male), n (%)**	33 (70.2)	32 (68.1)	0.823	0.046
**Baseline FMS, n (%)**			0.502	
4	0 (0.0)	1 (2.1)		0.147
5	1 (2.1)	2 (4.3)		0.105
6	46 (97.9)	44 (93.6)		0.174
**Tumor volume (cm^3), median (IQR)**	320 (182-449.3)	162.5 (59.3-361.7)	0.668	0.067
**Highest Level of Sacral Involvement, n (%)**			0.184	
S1	7 (14.9)	7 (14.9)		0.000
S2	20 (42.6)	13 (27.7)		0.333
S3	12 (25.5)	13 (27.7)		0.048
S4	8 (17.0)	8 (17.0)		0.000
S5	0 (0.0)	4 (8.5)		0.305
Coccyx	0 (0.0)	2 (4.3)		0.211
**Karnofsky Performance Scale, n (%)**			0.152	
100	1 (2.1)	3 (6.8)		0.186
90	32 (68.1)	26 (59.1)		0.183
80	9 (19.1)	14 (31.8)		0.272
70	5 (10.6)	1 (2.3)		0.561
Unknown	0	3		NA
**ECOG Performance Status, n (%)**			**<0.001**	
0	1 (2.1)	31 (68.9)		1.42
1	41 (87.2)	13 (28.9)		1.26
2	5 (10.6)	1 (2.2)		0.561
Unknown	0	2		NA
**Follow-up (months), Median (95% CI)**	68.1 (44.0-102.6)	58.6 (25.6-123.5)	0.572	0.095
**Overall survival (months), median (95% CI)**	68.1 (44.0-102.6)	58.6 (25.6-123.5)	0.572	0.095
**Progression-free survival (months), median (IQR)**	46.2 (33.3-75.7)	40.7 (18.1-82.3)	0.550	0.100
**Local recurrence, n (%)**	9 (19.1)	10 (21.3)	0.797	0.052
**Distant metastasis, n (%)**	14 (29.8)	12 (25.5)	0.645	0.098

© 2022 Yolcu YU et al. *JAMA Network Open.*

eTable 2. Characteristics of matched[¥] CIRT vs en bloc resection cohorts (cont.)

	CIRT (N=47)	En bloc Resection (N=47)	P-value	SMD
**Site of distant metastasis, n (% of metastasized tumors)**			0.747	
Spinal/pelvis bone	4 (28.6)	3 (25.0)		0.083
Lung	5 (35.7)	3 (25.0)		0.247
Other	5 (35.7)	6 (50.0)		0.286
**Peripheral motor neuropathy grade at last follow up prior to recurrence, n (%)***			**0.003**	
0	42 (89.4)	18 (52.9)		0.730
1	3 (6.4)	8 (23.5)		0.404
2	1 (2.1)	5 (14.7)		0.355
3	1 (2.1)	3 (8.8)		0.236
Unknown	0	13		NA
**Urinary retention, n (%)**			0.335	
No	31 (66.0)	20 (55.6)		0.209
Yes	16 (34.0)	16 (44.4)		0.209
Unknown	0	11		NA
**Colostomy, n (%)**	9 (19.1)	11 (23.4)	0.614	0.101
**Change in FMS (from baseline to last follow-up before recurrence), n (%)**			0.161	
Improved	0 (0.0)	1 (2.1)		0.153
Same	38 (80.9)	27 (57.4)		0.400
Worse	9 (19.1)	16 (34.0)		0.358
Unknown	0 (0.0)	3 (6.5)		NA
**Cause of death, n (%)**			NA	
Old age	2 (4.3)	NA		NA
Infectious disease	1 (2.1)	NA		NA
Cardiovascular disease	1 (2.1)	NA		NA
Pneumonitis	1 (2.1)	NA		NA
Other	1 (2.1)	NA		NA

CIRT: Carbon Ion Radiotherapy, FMS: Functional Mobility Scale, SMD: standardized mean difference
[¥]1:1 matching was performed for age, sex, baseline FMS, highest tumor level, tumor volume
*CTCAE version 4.03, NA: Not available

© 2022 Yolcu YU et al. *JAMA Network Open.*

eTable 3. Baseline characteristics of NCDB cohorts

	**Negative margins, No RT* (n=327)**	**Negative margins, Adjuvant RT (n=99)**	***Negative margins, +/- RT (n=426)***	**Positive margins, No RT (n=54)**	**Positive margins, Adjuvant RT (n=48)**	***Positive margins, +/- RT (n=102)***	**No surgery, Primary RT (n=141)**	**Surgery, any margins, +/- RT (n=528)**	**Surgery, any margins, no RT (n=381)**	**Surgery, any margins, Adjuvant RT (n=147)**
Age, median (IQR)	61 (50-70)	58 (47-67)	*61 (49-69.8)*	67 (52.3-73.5)	64.5 (52.8-72.3)	*66 (52.3-72.8)*	76 (65-82)	62 (50-71)	62 (50-71)	60 (48-69.5)
Sex (male), n (%)	205 (62.7)	67 (67.8)	*272 (63.8)*	31 (57.4)	35 (72.9)	*66 (63.5)*	72 (51.1)	338 (64.0)	236 (61.9)	102 (69.4)
Tumor size (mm)**, median (IQR)	70 (45-95)	70 (45-96)	*70 (45-95)*	100 (60-137.5)	72 (56.5-102.5)	*81 (57.8-120)*	81 (62.5-106.5)	71 (46-100)	70 (46-100)	72 (50-100)
Adjuvant chemotherapy, n (%)	4 (1.2)	4 (4.0)	*8 (1.9)*	1 (1.9)	3 (6.3)	*4 (3.9)*	13 (9.2)	12 (2.3)	5 (1.3)	7 (4.8)
Follow-up (months), Median (IQR)	56.3 (33.8-91.6)	62.9 (37.1-84.6)	*58.6 (35.0-65.0)*	60.6 (35.2-79.7)	51.6 (38.7-70.7)	*55.0 (37.3-73.1)*	33.0 (19.6-51.8)	58.1 (35.3-87.6)	56.4 (34.8-90.7)	60.2 (37.2-80.6)
Overall survival/Last contact (months), median (IQR)	56.3 (33.8-91.6)	62.9 (37.1-84.6)	*58.6 (35.0-65.0)*	60.6 (35.2-79.7)	51.6 (38.7-70.7)	*55.0 (37.3-73.1)*	33.0 (19.6-51.8)	58.1 (35.3-87.6)	56.4 (34.8-90.7)	60.2 (37.2-80.6)

*RT: radiotherapy
**maximal tumor diameter

© 2022 Yolcu YU et al. *JAMA Network Open.*

eTable 4. Characteristics and Overall Survival of matched CIRT cohort vs each NCDB cohort

	CIRT (n=188)	Negative margins, No RT* (n=188)	P-value	SMD	CIRT (n=99)	Negative margins, Adjuvant RT (n=99)	P-value	SMD
Age, median (IQR)	66 (58-71)	66 (57.8-71)	0.983	0.002	59 (51.5-67)	58 (47-67)	0.261	0.148
Sex (male), n (%)	128 (68.1)	123 (65.4)	0.584	0.057	68 (68.7)	67 (67.7)	0.879	0.022
Follow-up (months), Median (IQR)	62.2 (36.5-88.4)	59.0 (31.3-91.6)	0.220	0.016	67.7 (44.0-95.5)	62.9 (37.1-84.6)	0.760	0.196
Overall survival/Last contact (months), median (IQR)	62.2 (36.5-88.4)	59.0 (31.3-91.6)	0.220	0.016	67.7 (44.0-95.5)	**62.9** (37.1-84.6)	0.760	0.196
	**CIRT (n=188)**	**Negative margins, +/- RT (n=319)**	**P-value**	**SMD**	**CIRT (n=162)**	**Positive margins, No RT (n=54)**	**P-value**	**SMD**
Age, median (IQR)	66 (58-71)	65 (56-71)	0.260	0.016	67 (56.3-72.0)	67 (52.3-73.5)	0.889	0.020
Sex (male), n (%)	128 (68.1)	215 (67.4)	0.873	0.057	102 (63)	31 (57.4)	0.467	0.112
Follow-up (months), Median (IQR)	62.2 (36.5-88.4)	60.2 (32.7-89.3)	0.660	0.019	64.7 (38.5-88.5)	60.6 (35.2-79.7)	0.028	0.134
Overall survival/Last contact (months), median (IQR)	62.2 (36.5-88.4)	60.2 (32.7-89.3)	0.660	0.019	64.7 (38.5-88.5)	60.6 (35.2-79.7)	0.028	0.134
	**CIRT (n=144)**	**Positive margins, Adjuvant RT (n=48)**	**P-value**	**SMD**	**CIRT (n=102)**	***Positive margins, +/- RT (n=102)***	**P-value**	**SMD**
Age, median (IQR)	65 (56-71)	64.5 (52.8-72.3)	0.476	0.100	66 (53.3-71.8)	66 (52.3-72.8)	0.965	0.006
Sex (male), n (%)	105 (72.9)	35 (72.9)	1.00	0.000	70 (68.6)	66 (64.7)	0.552	0.082
Follow-up (months), Median (IQR)	64.7 (40.8-89)	51.6 (38.7-70.7)	0.110	0.373	71.8 (45.4-97.4)	55 (37.3-73.1)	0.012	0.422
Overall survival/Last contact (months), median (IQR)	64.7 (40.8-89)	51.6 (38.7-70.7)	0.110	0.373	71.8 (45.4-97.4)	55 (37.3-73.1)	0.012	0.422

© 2022 Yolcu YU et al. *JAMA Network Open.*

	CIRT (n=108)	No surgery, Primary RT (n=108)	P-value	SMD	CIRT (n=188)	Surgery, any margins, +/- RT (n=376)	P-value	SMD
Age, median (IQR)	71 (63-75.3)	72.5 (62.5-78)	0.484	0.090	66 (58-71)	65 (57-71)	0.589	0.048
Sex (male), n (%)	61 (56.5)	66 (61.1)	0.489	0.093	128 (68.1)	249 (66.2)	0.658	0.040
Follow-up (months), Median (IQR)	64.9 (40.3-86.9)	31.8 (18.2-51.3)	<0.001	0.987	62.2 (36.5-88.4)	57.6 (35.1-87.2)	0.230	0.061
Overall survival/Last contact (months), median (IQR)	64.9 (40.3-86.9)	31.8 (18.2-51.3)	<0.001	0.987	62.2 (36.5-88.4)	57.6 (35.1-87.2)	0.230	0.061
	**CIRT (n=188)**	**Surgery, any margins, no RT (n=299)**	**P-value**	**SMD**	**CIRT (n=129)**	**Surgery, any margins, Adjuvant RT (n=129)**	**P-value**	**SMD**
Age, median (IQR)	66 (58-71)	66 (54-71.5)	0.232	0.031	63 (55-71)	63 (53-71)	0.691	0.042
Sex (male), n (%)	128 (68.1)	204 (68.2)	0.974	0.011	87 (67.4)	87 (67.4)	1.000	0.000
Follow-up (months), Median (IQR)	62.2 (36.5-88.4)	53.4 (31.9-87.8)	0.130	0.065	66.1 (43.6-94.3)	58.9 (37-76.8)	0.400	0.323
Overall survival/Last contact (months), median (IQR)	62.2 (36.5-88.4)	53.4 (31.9-87.8)	0.130	0.065	66.1 (43.6-94.3)	58.9 (37-76.8)	0.400	0.323

*RT: radiotherapy

© 2022 Yolcu YU et al. *JAMA Network Open.*

eFigure 5. Kaplan-Meier plots comparing OS between matched¥ CIRT and primary radiotherapy NCDB cohorts

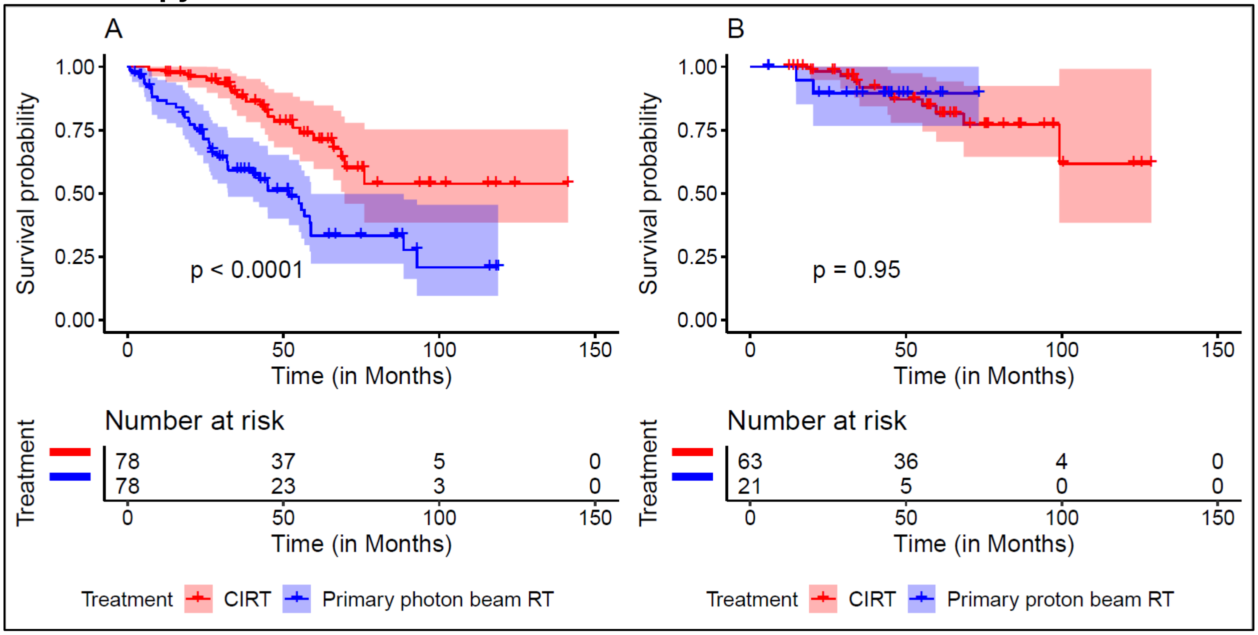

eFigure 5A displays patients treated with primary photon radiotherapy (RT); eFigure 5B displays patients treated with primary proton radiotherapy (RT).

¥1:1 matching was performed for age and sex in eFigure 5A. 3:1 matching was performed for age and sex in eFigure 5B.

© 2022 Yolcu YU et al. *JAMA Network Open.*

eTable 5. Radiotherapy treatment factors by modality for unmatched NCDB patients who underwent primary radiotherapy (RT) and for primary CIRT* patients

	CIRT (n=188)	Photon RT (n=80)§	Proton RT (n=30)¶	P-value
Age, median (IQR)	66.0 (58.0-71.0)	76.0 (66.8-82.0)	76.0 (65.3-81.8)	
Sex (male), n (%)	128 (68.1)	35 (43.8)	14 (46.7)	
Number of fractions, median (IQR)	16 (16-16)	28 (5-33.0)	42 (41-42)	<0.001
Total dose (Gy)**, median (IQR)	67.2 (67.2-70.4)	58.5 (30-67.1)	75.6 (70.4-76.1)	<0.001
BED (alpha/beta 2.0¥), Median (IQR)	208.3 (208.3-225.3)	137.5 (120-156.1)	143.6 (133.8-145.1)	<0.001

*CIRT: carbon ion radiotherapy
§78 patients had radiotherapy data available for review
¶21 patients had radiotherapy data available for review
**RBE 3.0 for CIRT, RBE 1.1 for protons
¥Radiat. Oncol. 2014; 9: 100. Published online 2014 Apr 29. doi: 10.1186/1748-717X-9-100. PMCID: PMC4016619. PMID: 24774721

eTable 5 shows radiotherapy details for patients who underwent primary photon or proton radiotherapy only. However, patients in the NCDB cohort treated with photon therapy alone were treated to a relatively low dose which prevented an adequate comparison to protons and CIRT. In the small group of proton patients available in our study, the direct comparison to CIRT did not reveal any significant difference in overall survival recognizing, however, that the biologically effective dose (BED) used for the CIRT patients was significantly higher than the proton patients. Despite the small number of patients, the outcomes for CIRT should theoretically be superior to proton radiotherapy due to the higher linear energy transfer (LET), lower oxygen enhancement ratio (OER), and higher relative biological effectiveness (RBE). However, when controlling for BED, there was still no difference in OS. Overall survival may not be a good surrogate for local tumor control because there may be a difference in local tumor control which is not reflected in OS. In contrast, patients undergoing radiotherapy alone with photons had a lower overall survival compared to the CIRT group ($p<0.001$), even when controlling for BED ($p<0.001$) suggesting a possible LET, OER, and RBE advantage to CIRT. The data from this study is being used to design a prospective trial sponsored by Mayo Clinic comparing outcomes for patients treated with CIRT or proton therapy.

© 2022 Yolcu YU et al. *JAMA Network Open.*

It is also theorized that these potential advantages of CIRT may be overcome through hypofractionation of protons. There is an ongoing Phase I/II trial comparing proton radiotherapy to CIRT in a population of patients with evidence of macroscopic tumor [Ion Irradiation of Sacrococcygeal Chordoma (ISAC), NCT01811394].[1] This study allows patients treated with both radiotherapy alone and margin-positive surgery with adjuvant radiotherapy. However, separate evaluations for the two groups would be a better comparison. In addition, there are concerns that the trial design may not allow adequate evaluation of the outcomes due to the short 3-year tumor control and survival endpoint, the relatively low CIRT dose of 64 Gy (RBE3.0) and using different methods of estimating RBE in the CIRT treatment planning systems.

© 2022 Yolcu YU et al. *JAMA Network Open.*

eAppendix 2. Cost Analysis

Two-year standardized Medicare costs were available for 28 patients treated with en bloc resection and 15 patients treated with proton radiotherapy at Mayo Clinic (**eTable 6**). Both mean total costs and total procedural costs were significantly higher for the en bloc resection cohort at $137,182 (SD $117,894) and $110,375 (SD $94,169) versus $68,066 (SD $60,567) and $53,699 (SD $43,052) for the proton group, p=0.02 and p<0.01 respectively. Proton radiotherapy costs at Mayo Clinic of $30,489 (SD $4,530) were comparable to CIRT costs at QST Hospital of $22,800. This equates to an estimated price per fraction of $924 for proton radiotherapy (33 fraction course) and $1,425 for CIRT (16 fraction course).[2] An estimate for possible U.S. Medicare reimbursement per fraction of CIRT was calculated to be $1,906 per fraction assuming price parity between courses of proton radiotherapy and CIRT treatment episodes.

Despite the small numbers of patients treated comparatively with proton radiotherapy, OS was similar to patients treated with CIRT, and both were superior to primary photon radiotherapy. Our cost analysis shows that CIRT total and procedural costs in the U.S. are likely to be on par with that of proton radiotherapy. While the theoretical benefits of LET, OER, and RBE of CIRT may result in improved local control, although perhaps not OS, ongoing and future research studies are vital to understanding these differences and improving radiotherapy modalities.

© 2022 Yolcu YU et al. *JAMA Network Open.*

eTable 6. Two-year standardized Medicare costs for en bloc resection and proton radiotherapy at Mayo Clinic from 2007 to 2016

	Proton Radiotherapy (N=15)		En bloc resection (N=28)			
**Cost Category**	**Mean**	**Standard Deviation**	**Mean**	**Standard Deviation**	**Difference (%)**	**P-value[1]**
E&M	$1,601	$2,694	$5,286	$4,596	230%	*<0.01*
Imaging	$3,428	$2,230	$9,128	$7,450	166%	*<0.01*
Other	$3,336	$5,045	$12,393	$13,800	271%	*<0.01*
Radiotherapy	$30,489	$4,530	$0	$0		*<0.01*
Procedures	$6,003	$8,763	$23,684	$20,143	295%	*<0.01*
Hospital Services	$23,209	$42,942	$86,691	$74,779	274%	*<0.01*
Total Procedural Costs[¥]	$53,699	$43,052	$110,375	$94,169	106%	*0.02*
Total Two-Year Costs	$68,066	$60,567	$137,182	$117,894	102%	*0.02*

E&M: Evaluation and Management. Other: Includes emergency department, chemotherapy, labs, and otherwise uncategorized costs.
All cost categories are two-year, standardized Medicare costs adjusted to inflation to 2017.
[1]Mann-Whitney U Test. [¥]Sum of Procedure, Hospital Services, and Radiotherapy Costs.

© 2022 Yolcu YU et al. *JAMA Network Open.*

eReferences

1. ClinicalTrials.gov. Ion Irradiation of Sacrococcygeal Chordoma. 2018. https://www.clinicaltrials.gov/ct2/show/NCT01811394

2. Ministry of Health, Labour, and Welfare. Outline of FY2016 Revision of Medical Service Fees (DPC System-Related Section). 2016. https://www.mhlw.go.jp/file/06-Seisakujouhou-12400000-Hokenkyoku/0000115023.pdf

© 2022 Yolcu YU et al. *JAMA Network Open.*
